# Water memory effects and their impacts on global vegetation productivity and resilience

**DOI:** 10.1038/s41598-018-21339-4

**Published:** 2018-02-13

**Authors:** Laibao Liu, Yatong Zhang, Shuyao Wu, Shuangcheng Li, Dahe Qin

**Affiliations:** 1 College of Urban and Environmental Sciences, Peking University, Beijing, 100871 China; 2 Key Laboratory for Earth Surface Processes of The Ministry of Education, Peking University, Beijing, 100871 China; 3 State Key Laboratory of Cryospheric Sciences, Cold and Arid Regions Environmental and Engineering Research Institute, Chinese Academy of Sciences, Lanzhou, China

## Abstract

Memory effects refer to the impacts of antecedent climate conditions on current vegetation productivity. This temporal linkage has been found to be strong in arid and semi-arid regions. However, the dominant climatic factors that determine such patterns are still unclear. Here, we defined’water-memory effects’ as the persistent effects of antecedent precipitation on the vegetation productivity for a given memory length (from 1 to up to 12 months). Based on satellite observations and climate data, we quantified the length of water-memory effects and evaluated the contributions of antecedent precipitation on current vegetation. Our results showed that vegetation productivity was highly dependent on antecedent precipitation in arid and semi-arid regions. The average length of water memory was approximately 5.6 months. Globally, water-memory effects could explain the geographical pattern and strength of memory effects, indicating that precipitation might be the dominant climatic factor determining memory effects because of its impact on water availability. Moreover, our results showed vegetation in regions with low mean annual precipitation or a longer water memory has lower engineering resilience (i.e. slower recovery rate) to disturbances. These findings will enable better assessment of memory effects and improve our understanding of the vulnerability of vegetation to climate change.

## Introduction

Memory effects can be defined as the dependence of vegetation productivity on both contemporary disturbances and the residual effects of past climate conditions^1–4^. The occurrence of such effects highlights the importance of considering time in ecological studies and provides an explanation for the observed relationship between antecedent climate conditions and current vegetation growth^2^. Indeed, there is clear evidence that past climate conditions, including precipitation, temperature, and other abiotic factors can significantly influence phenology^5,6^, vegetation productivity^7–11^ and terrestrial ecosystem carbon cycles^12,13^. However, the relationship between past climate conditions and current vegetation productivity is still poorly understood^2^. Hence, a better understanding of vegetation memory effects is a critical requirement for projecting future vegetation dynamics.

Recent studies have indicated that memory effects play a more significant role in influencing the vegetation productivity of arid and semi-arid regions at a global scale^1,4^. However, the first-order autoregressive [AR (1)] model adopted in previous studies^1,4^ have not identified the relative contributions of each climatic factor to the observed effects. Thus, the underlying dominant climatic driver that explains this pattern remains unknown, despite the knowledge that vegetation in arid and semi-arid regions is predominantly limited by water availability^14^. Additionally, although the effects of antecedent precipitation on vegetation growth in dry regions have been established in previous studies^7,9,11,15–18^, there has been a lack of analysis of the contributions of antecedent water availability to memory effects globally. The elucidation of such contributions should provide valuable insight into the mechanism of global vegetation memory effects and contribute to a more comprehensive understanding of water-related vegetation responses. Here, we defined vegetation ‘water-memory effects’ as the persistent effects of antecedent precipitation on the vegetation productivity for a given memory length (from 1 to up to 12 months). Since the time lag of the responses of vegetation to antecedent precipitation differ significantly^19,20^, an important approach for evaluating water memory effects is to determine the period during which past precipitation significantly influenced the current state of vegetation productivity, which as can be referred to as ‘water-memory length’^2^.

In addition, memory effects are considered to be a useful indicator for quantifying the recovery rates of natural ecosystems to their equilibrium state after perturbations from fluctuations in climatic or other factors, which is an important component of ecosystem engineering resilience^4,21^. Specifically, engineering resilience estimates the recovery rates of a system to equilibrium state after the disturbance^22^, whereas ecological resilience quantifies the magnitude of disturbances that could be absorbed by a system changes to another state^23^. Studies indicate that systems with lower ecological resilience may also have longer ecological memory and therefore may also indicate slower recovery rates (i.e. lower engineering resilience)^24^. This slowing of ecosystem recovery can be detected through an increase in temporal autocorrelation^4,24,25^. For instance, Verbesselt *et al*.^25^, adopted this method using satellite observations and found that tropical forests in drier regions exhibit slower recovery rates and therefore inferred that these areas had lower ecological resilience. This finding suggested that precipitation could be an important mechanism underlying tropical forest resilience. Therefore, precipitation and the memory length may be critical for understanding vegetation engineering resilience and could be used to help recognize ecosystems that are vulnerable to collapse.

In this study, climate-vegetation relationships were determined using multiple linear regression (MLR) models and one AR (1) model, employing use of 31 years of satellite-derived Normalized Difference Vegetation Index (NDVI) data^26^ (a proxy of vegetation productivity) and the Standardized Precipitation Index (SPI) together with global precipitation, temperature and solar radiation data (See Methods). Here, we aim to (1) determine the period during which past precipitation significantly influenced the current state of vegetation productivity, i.e., water memory length; (2) evaluate the contributions of within-memory precipitation (i.e., accumulated precipitation within the water memory length) to memory effects and investigate the reason why stronger memory effects are observed in global arid and semi-arid regions; and (3) investigate the relationship between precipitation/water memory length and ecosystem engineering resilience at the global scale to improve our understanding of vegetation resilience.

## Results

### Length of water memory effects

**Figure 1:**
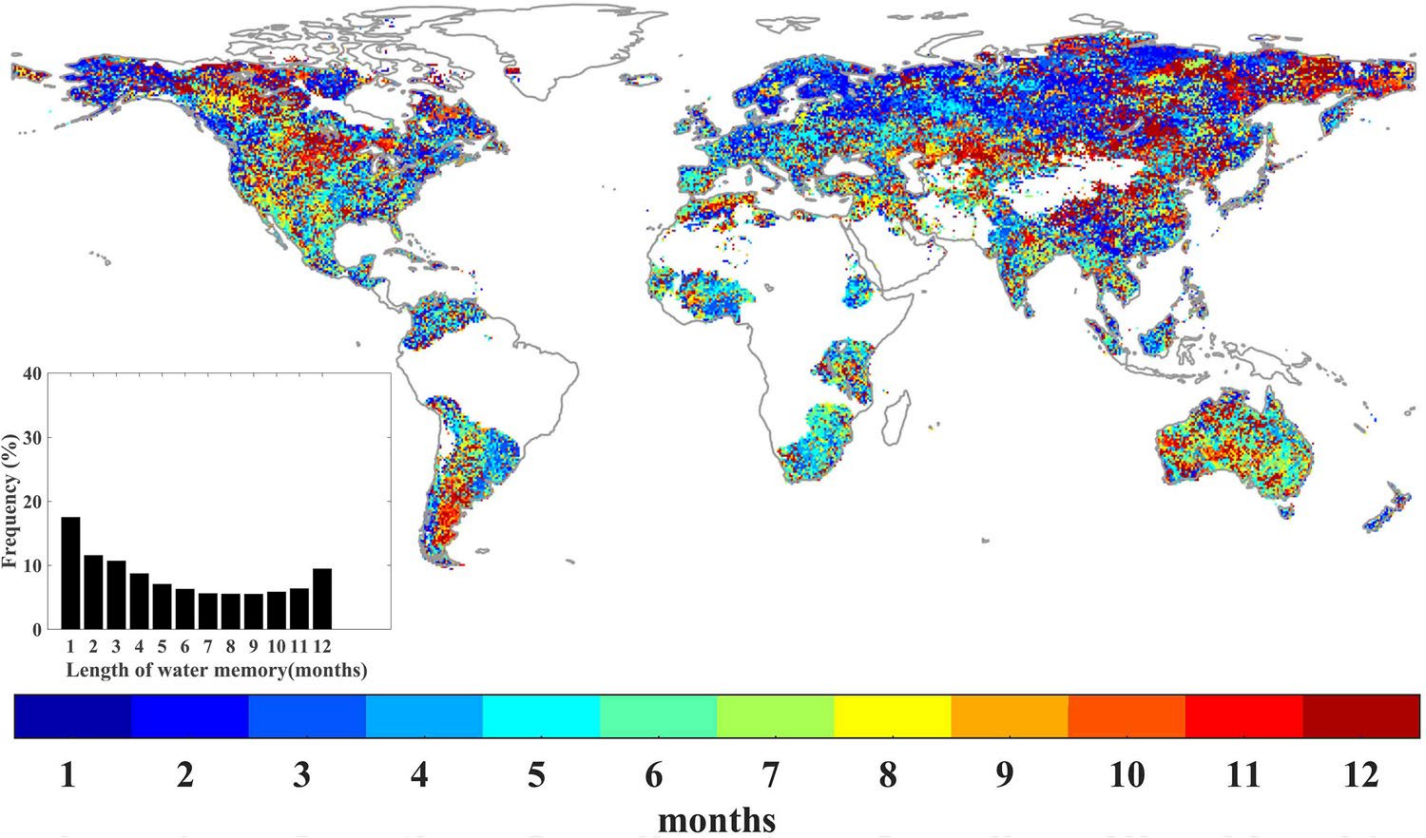
Spatial distribution of the length of water memory during the growing season, from 1982 to 2012, on a monthly basis. Areas with barren land (mean NDVI < 0.1 for all months), permanent ice, and the percentage of missing values greater than 5% in the CRU TS4.01 climate datasets are not shown. The inset shows the frequency distribution of water memory length. NDVI, Normalized Difference Vegetation Index. This map was produced using MATLAB R2016b (http://www.mathworks.com/products/matlab/).

On a global scale, we first determined the water memory length as the period for which the correlation coefficient between NDVI and within-memory precipitation (SPI) was highest (See Methods). Note that we removed all areas where the percentage of missing values in the Climate Research Unit (CRU) climate datasets was greater than 5% based on the station files (Fig. S1). We found that NDVI in 72.8% of the vegetated land areas was significantly correlated with SPI (P < 0.05) (Fig. S2), especially in arid and semi-arid regions of vegetated areas, as identified by the aridity index (Fig. S3a). Water memory length during the growing season exhibited high spatial heterogeneity but mainly ranged from 1 to 3 months (Fig. 1). We also found that longer memory was prominent in several arid and semi-arid regions, including central-western America, southern Argentina, western Australia, and central Asia.

In addition, based on a global unchanged land cover map that was derived from moderate-resolution imaging spectroradiometer (MODIS) data (Fig. S4), we found that southern open shrubland and grassland exhibited the greatest water memory length (6.9 and 6.5 months, respectively). Additionally, forest and savanna ecosystems presented shorter water memory lengths than other vegetation types. The mean lengths of the water memory effects for deciduous broadleaf, evergreen broadleaf, deciduous needleleaf, and evergreen needleleaf forests were 5.2, 5.0, 5.2, and 4.5 months, respectively (Table S1). Furthermore, we analyzed the time scales at which the correlation coefficients between vegetation productivity and within-memory precipitation were highest at a monthly resolution and found that only a few areas exhibited significant relationships during each month of the main growing season, such as Australia (Fig. S5).

### Contribution of precipitation to memory effects

The water memory length is the length of time over which past precipitation significantly impacts the current vegetation productivity. Therefore, to quantify the effects of variation in within-memory precipitation on NDVI during the growing season from 1982 to 2012, we used a MLR model in which NDVI was the dependent variable and the independent variables were as follows: SPI at the timescale of water memory length, temperature, and solar radiation (cloud cover as the proxy). Accordingly, the linear regression slope of SPI to NDVI (i.e., the SPI coefficient) was defined as the effects of within-memory precipitation on NDVI, and the influence of contemporary temperature and solar radiation were quantified in the same manner. For a comparison, we also constructed a MLR model without considering antecedent precipitation, in which SPI was replaced by the contemporary precipitation. The variables were all detrended and standardized. In addition, we constructed an AR (1) model to characterize the strength of the general vegetation memory effects (i.e., the coefficient of NDVI_t−1_) when antecedent water availability is not considered^1,4^(Equation 3, see Methods).

**Figure 2:**
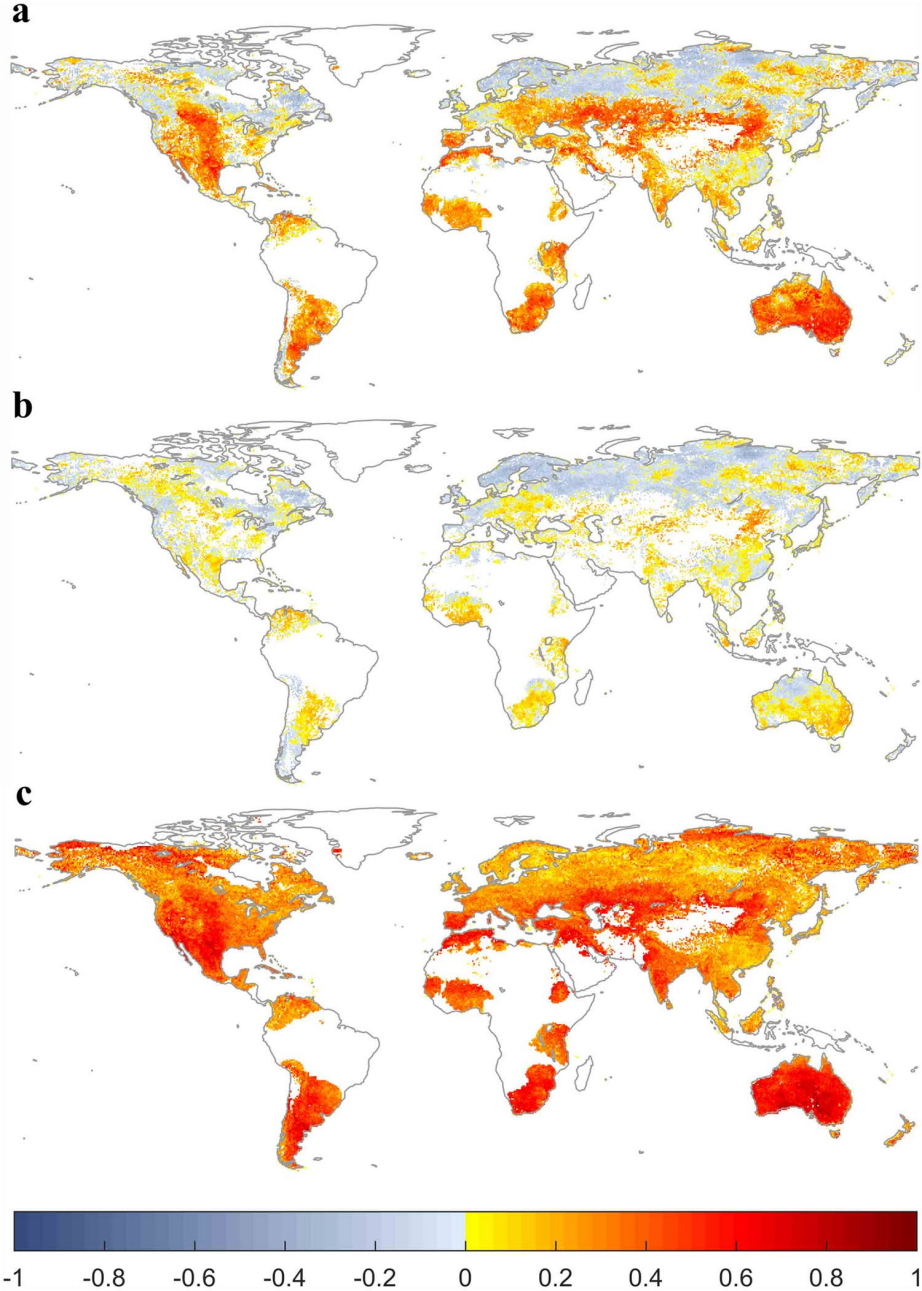
Role of within-memory precipitation in determining global memory effects. (**a**) Sensitivity of NDVI to within-memory precipitation (coefficient of SPI, from MLR model considering antecedent precipitation). (**b**) Sensitivity of NDVI to contemporary precipitation (coefficient of P, from MLR model without considering antecedent precipitation). (**c**) Sensitivity of NDVI to NDVI_t−1_ (coefficient of NDVI_t−1_, from AR1 model). Areas with no significant relationship (P > 0.05), barren land (mean NDVI < 0.1 for all months), permanent ice, and the percentage of missing values greater than 5% in the CRU TS4.01 climate datasets are not shown. Maps were produced using MATLAB R2016b (http://www.mathworks.com/products/matlab/).

**Figure 3:**
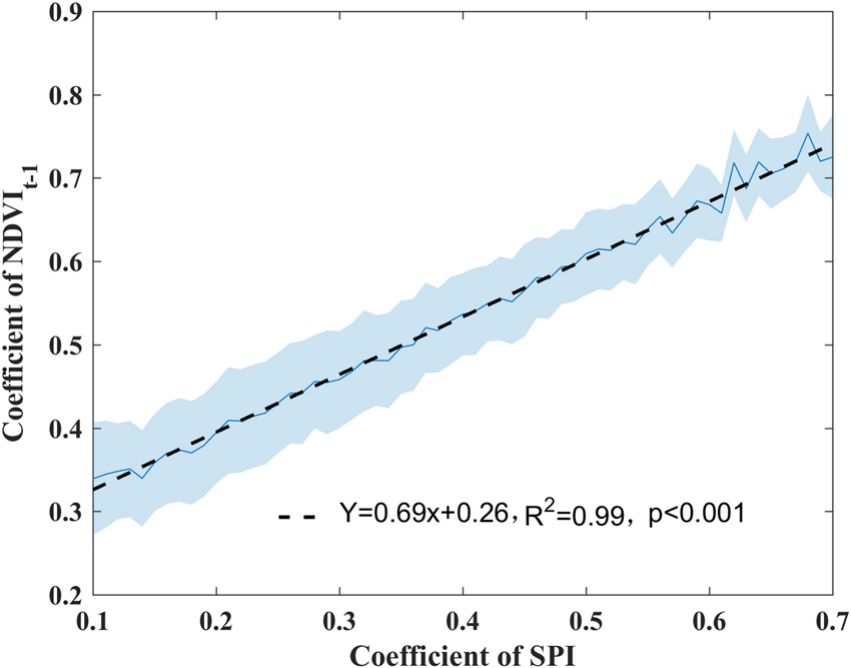
Relationship between the coefficients of the standardized precipitation index (SPI) and the normalized difference vegetation index (NDVI_t−1_). Both variables are detrended and standardized for the corresponding period. Note that the values of the SPI coefficient characterize the sensitivity of NDVI to variation in SPI. The solid line and shaded area represent the means ± SD/2. The dashed line represents the linear regression of the SPI coefficients from the MLR model and the NDVI_t−1_ coefficients from the AR (1) model.

First, we compared the average explanatory power of the three climatic factors for vegetation growth with or without considering water memory effects. We found that the MLR model with water memory effects provided a better explanation for the variation in vegetation productivity globally (Fig. S6). Furthermore, vegetation in arid and semi-arid regions showed much higher sensitivity (higher SPI coefficient values) to water availability when antecedent precipitation was considered than when it was not (Fig. 2a and b). More importantly, this result is consistent with areas of strong memory effects (high NDVI_t−1_ coefficient values) (Fig. 2a and c). To determine the strength of the linkage between water-memory effects and the general memory effects from the AR (1) model, we analyzed the correlation between the SPI and NDVI_t−1_ coefficients and found a particularly strong correlation (R^2^ = 0.99, p < 0.001) (Fig. 3), which indicated that water memory effects could dominate the strength of memory effects, especially in arid and semi-arid regions. Additionally, weak water memory effects were also observed at middle-to-high latitudes in the Northern Hemisphere and tropical forests, where the climate is more humid (Fig. 2a). To test the dependency of the strength of water memory effects on the gradient of precipitation, the pixel values of SPI coefficient were averaged by MAP bin (each interval size of 50 mm yr^−1^). As illustrated in Fig. S7, the coefficients of SPI decreased with increasing MAP (R^2^ = 0.89, P < 0.001). Furthermore, similar results were also observed when we used other precipitation datasets from Global Precipitation Climatology Centre^27^ and University of Delaware^28^, a temperature dataset from University of Delaware and a CRU-NCEP shortwave radiation dataset (Figs S8–12).

In addition, weak memory effects (low NDVI_t−1_ coefficient values) were mainly observed at middle-to-high latitudes in the Northern Hemisphere and tropical forests (Fig. 2c), where temperature and shortwave radiation are the predominant limiting climatic factors^1,14^. Positive coefficients of temperature were also observed in these regions (Figs S13a–15a). Furthermore, we also observed negative coefficients of temperature in Australia and Africa, which indicated that the NDVI anomalies in these regions respond in an opposite manner, which may might suggest a co-effect of water limitation^29,30^. Additionally, the coefficient of solar radiation was smaller than those of the other two climatic factors, which implied that NDVI was less sensitive to variation in solar radiation than to either precipitation or temperature. However, the sensitivity of vegetation productivity to variation in solar radiation was higher at high latitudes in the Northern Hemisphere, as well as in central Europe, southern China, and northwest Australia (Figs S13b–15b). Nevertheless, no general pattern was found in the responses of vegetation to the effects of radiation variability.

### Relationship between precipitation/water memory length and vegetation engineering resilience

According to a previous study^4^, the AR(1) model (equation 3) used to denote general memory effects globally may be used as a proxy for vegetation engineering resilience. Specifically, high absolute values of the coefficient of NDVI_t−1_ (γ) may indicate a slow recovery rate to equilibrium, i.e., low engineering resilience^4^. Therefore, it may be interesting to assess the relationship between precipitation/water memory length and vegetation engineering resilience here.

**Figure 4:**
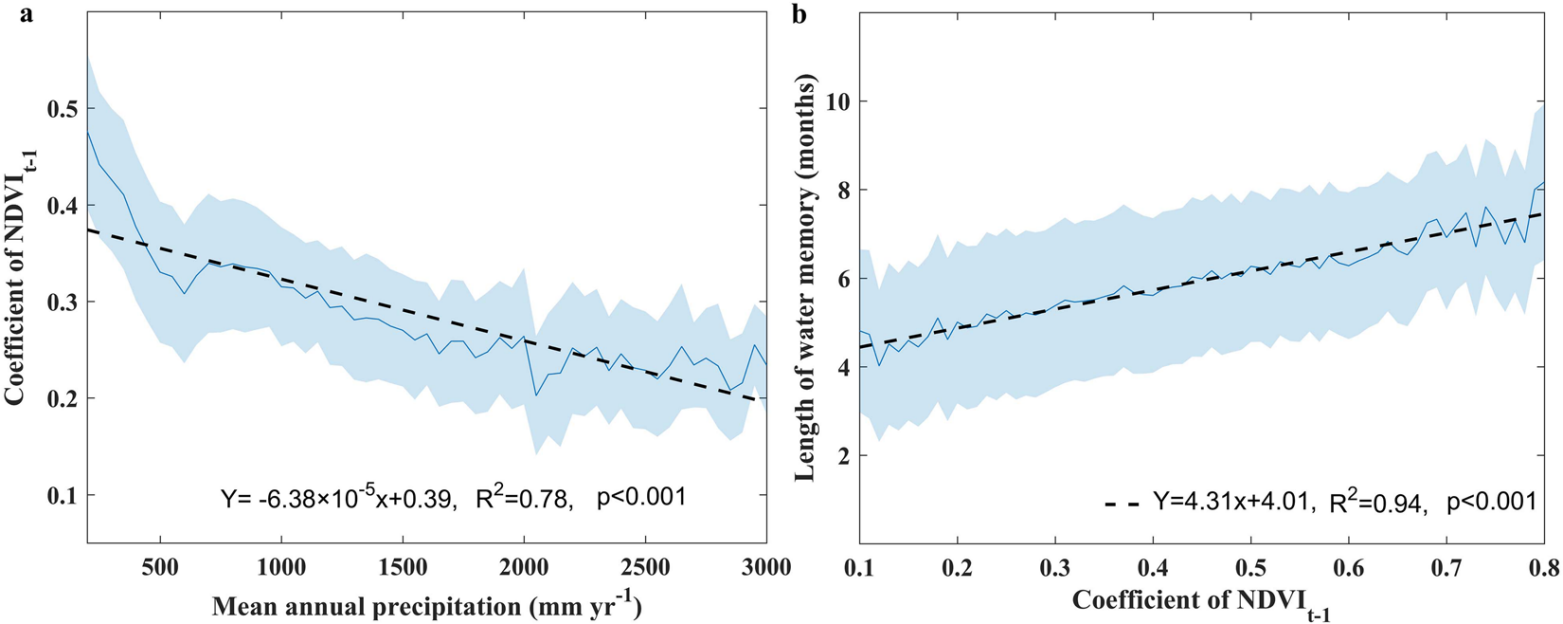
Relationship between the recovery rate and (**a**) MAP and (**b**) the length of water memory. All variables are detrended and standardized for the corresponding period. High coefficient of NDVI_t−1_ indicates the low recovery rate. The solid line and shaded area represent the means ± SD/2. The dashed line represents the linear regression of NDVI_t−1_ coefficient from the AR (1) model and MAP in a. The dashed line represents the linear regression of the length of water memory and the NDVI_t−1_ coefficient in b.

Based on the analysis of the AR (1) models (Fig. 2c), we found that the coefficients of NDVI_t−1_ increased markedly when the MAP fell globally (Fig. 4a). Among different vegetation types, the highest coefficient of NDVI_t−1_ was observed in southern open shrubland (0.59) where the lowest MAP was observed (318 mm). In contrast, forests appeared to exhibit low coefficients of NDVI_t−1_. For example, evergreen broadleaf forest presented the lowest coefficient of NDVI_t−1_ (0.25), with the highest MAP (2352 mm) (Table S2). However, this relation is not strictly true among all vegetation types, such as savanna. In addition, as within-memory precipitation has been suggested to play a dominant role in determining memory effects (Figs 2a,c and 3), vegetation with a long water memory would likely to be out of equilibrium with contemporary climate perturbations. It might also be expected to exhibit slower recovery rates and lower ecological resilience. Indeed, on a global scale, we found a significant positive correlation between the coefficients of NDVI_t−1_ and the length of water memory (R^2^ = 0.94, p < 0.001) (Fig. 4b). For example, southern open shrubland displayed a long water memory length (6.9 months) and high coefficient of NDVI_t−1_ (low engineering resilience). Evergreen broadleaf forest have short water memory length (5.0 months) and low coefficient of NDVI_t−1_ (high engineering resilience). Spatially, several patterns were evident. For example, lower and higher engineering resilience levels were observed in southern and northern Argentina, respectively. A similar pattern was also observed for the MAP and the length of water memory; i.e., lower MAP and longer water memory were observed in southern Argentina, and higher MAP and shorter water memory were observed in northern Argentina (Fig. 1, Fig. 2c and Fig. S3b). Similar patterns were also apparent in eastern and western America. However, the patterns in several regions were less evident, such as South Africa.

## Discussion

In this study, we quantified the length of water memory effects and the contributions of within-memory precipitation to current vegetation productivity globally and implications of MAP and water memory length for vegetation engineering resilience.

### Importance of water memory effects

Our results concerning the vegetation water memory length suggested that arid and semi-arid regions showed the longest time scales, of approximately 5–7 months. The obtained time scales were comparable to a previous study that reported vegetation in arid and semi-arid regions were most sensitive to water anomalies for about 4–6 months^16^. Twelve-month-persistent responses of vegetation’s to antecedent precipitation were also identified in arid regions, such as central-western America and central Asia. This is supported by recent studies indicating that above-ground net primary production in arid ecosystems is highly dependent on previous-year precipitation^9,11^. This finding can be explained by (1) the adaptations of vegetation to frequent water shortages due to relevant physiological mechanisms;^20,31^ (2) the lagged vegetation response to soil moisture anomalies in arid or semi-arid regions;^3,32,33^ and (3) the hierarchical of responses of vegetation’s responses to resource availability^34^. Hence, it has been suggested that a hierarchical response framework should be be involved in future research to understand the complex mechanisms^11,35^.

Furthermore, we found that the strength of vegetation water memory effects was greatest in arid and semi-arid regions at the global scale and decreased with increasing MAP. This observation is consistent with previous studies, which have reported arid or semi-arid ecosystems showed largest legacy or time-lag effects of precipitation^15–17,19^. For instance, a recent investigation of the responses of forests to pervasive water deficits indicated that the legacy effects of water deficits on forest trees are highly dependent on MAP, with this effect being most pronounced in dry ecosystems^36^. Moreover, recent studies^1,4^ have shown that strong memory effects are found in arid and semi-arid regions globally. However, the AR (1) model adopted in previous studies^1,4^ cannot differentiate the dominant climatic factors determining memory effects globally. Our results showed that within-memory water availability can explain the geographical pattern and strength of memory effects globally. This suggests that antecedent precipitation may be the dominant climate factor that determine the memory effects, especially in arid and semi-arid regions.

In addition, many studies may underestimate the real sensitivity of vegetation to precipitation variability due to considering only the effects of short-term concurrent precipitation perturbations on vegetation growth^19,37^. This potential issue is supported by our finding that current vegetation productivity is more strongly affected by antecedent precipitation than contemporary precipitation in arid and semi-arid regions; i.e., vegetation shows the higher sensitivity of vegetation to within-memory precipitation than to contemporary precipitation. For example, Sala *et al*.^11^, found antecedent precipitation largely controlled current-year Aboveground Net Primary Production (ANPP) mainly in North America and central Asia based on 16 long-term *in situ* series of ANPP. This observation is consistent with our finding that vegetation in North America and central Asia shows higher sensitivity to within-memory precipitation than to contemporary precipitation. Hence, it is important to recognize that water-limited regions might also exhibit high sensitivity to climate variability and may therefore also be vulnerable to climate change^38^. In addition to the important role of antecedent precipitation in determining current vegetation dynamics, other factors, such as changes in antecedent temperature and solar radiation, could modify vegetation responses, as well^6,8^. Thus, there is also a compelling need to understand the temporal linkages between other abiotic factors, including temperature and shortwave radiation, and the ecological dynamics of current vegetation^2^.

### Implications for vegetation resilience

Another finding of this study was a linear relationship between the MAP and recovery rates, as may be inferred by the general AR (1) coefficient in equation 3. Due to the lack of very long time series of data that can describe a period of gradual change in the vegetation state^39^, we didn’t detect the changes in recovery rates across time nor assess the spatial patterns of it. This result of the declining recovery rates across the decreasing gradient of MAP is consistent with a previous study^4^ showing that vegetation in dry regions exhibits low engineering resilience at the global scale. A recent study also indicated that tropical forests in drier regions present slower recovery rates and therefore might be less resilient^25^. The declining recovery rates observed across the decreasing MAP gradient could be caused by the intrinsic differences in vegetation structures, such as tree cover and canopy height, or other mechanisms influenced by the variations in MAP^25^. For instance, Hirota *et al*.^40^, also found tropical forest show low resilience where there is low tree cover (low MAP)^40^, which may partly explained our estimates of low engineering resilience for savanna and higher engineering resilience for evergreen broadleaf forest. Moreover, as within-memory precipitation was suggested to play a dominant role in memory effects, vegetation with a greater water memory length is likely to recover to an equilibrium state more slowly, which is consistent with current theory^24^. However, vegetation resilience is complex and challenging to assess because it can be regulated by a number of mechanisms, including ecosystem biodiversity^41,42^, ecosystem structure^4,40^, and plant trait diversity^43^. It should be also noted that a high _t−1_ coefficient in the AR (1) model might be caused by some mechanisms other than low engineering resilience. For example, we identified that some regions with low engineering resilience actually responded to a lagged extrinsic water variable, which might be related to “extrinsic ecological memory”^2^. The next challenge is to understand the complex biological and hydrological processes underlying these patterns and relationships. Hence, in the future, long-term field experiments along the gradient of precipitation may help validate and clarify the mechanisms of vegetation engineering resilience based on remotely observations.

## Conclusions

In summary, based on the simultaneous analysis of satellite observations and climatic data, we found that the vegetation water memory length was prominent in arid and semi-arid regions, with a globally averaged water memory length of 5.6 months. Our results also concluded that water memory effects could explain the geographical pattern and strength of memory effects, suggesting that precipitation might be the dominant climatic factors determining memory effects because of its impact on water availability. This finding contributes to a more comprehensive understanding of water-related vegetation responses. In terms of the relationship between MAP/water memory length and vegetation engineering resilience, strong linear correlations were observed at a global scale. This finding may imply that vegetation in regions with low MAP or a longer water memory is likely to have low engineering resilience (i.e. slower recovery rate) to disturbances. Future field experiments are needed to confirm and interpret the satellite-derived vegetation engineering resilience results. Since future increases in the occurrences or intensity of extreme events are likely to generate unknown effects on terrestrial ecosystems and ecosystem services^44^, the assessment of vegetation water memory effects and engineering resilience can improve our understanding of the vulnerability of vegetation to climate change.

## Methods

### Climate data

We first used monthly precipitation, temperature and cloud cover datasets from the Climate Research Unit, version TS 4.01. These datasets had been interpolated from meteorological stations, based on spatial autocorrelation functions, and included data from 1901 to 2016^45,46^. The cloud cover has been widely used a proxy of solar radiation in previous studies^1,47^. Since the CRU dataset included fewer observations in the early part of its record (before the 1950s)^48^, we used only CRU precipitation dataset for 1960–2013 and the temperature and cloud cover datasets for 1982–2012. In addition, we used two precipitation datasets (Global Precipitation Climatology Centre (GPCC) version 7^27^ and (University of Delaware) UDel precipitation version 4^28^) and the new temperature dataset UDel version 4 as well as a new shortwave radiation dataset. The new shortwave radiation data for the same period were obtained from the CRU-NCEP dataset, version V5.2, which combined the CRU climate dataset from 1901 to 2012 and the NCEP reanalysis from 1948 to 2012. These climate datasets have a spatial resolution of 0.5° and temporal resolution of one month. These climate datasets were used separately to test the robustness of our results. A global aridity index dataset with a spatial resolution of 0.0083° from Consultative Group for International Agricultural Research Consortium for Spatial Information (CGIAR-CSI) was used to identify arid and semi-arid regions (Fig. S3a)^49,50^. All the climate datasets cover our study period of 1982 to 2012, and their use in global climate and vegetation studies is well established^20,47,51^.

### SPI data

Following a previous study^16^, we used the standard precipitation index (SPI) to quantify accumulated monthly precipitation anomalies. SPI is a traditionally multi-scale drought index that is useful as an indicator of cumulative precipitation anomalies. Negative SPI values indicate dry conditions, whereas positive values indicate wet conditions. Using the CRU, GPCC and UDel monthly precipitation data for 1960 to 2013, we calculated SPI from 1982 to 2012 at time scales from 1 to 12 months, following McKee *et al*.^52^.

### Satellite NDVI data

The third-generation Normalized Difference Vegetation Index (NDVI3g) data used in the present study were obtained from the Global Inventory Monitoring and Modelling Studies (GIMMS) group and were derived from the NOAA/AVHRR land dataset, at a 0.083° spatial resolution and 15-day interval, for the period of January 1982 to December 2012. The GIMMS3g NDVI dataset, which provides the longest time series, has been widely used for vegetation monitoring^18,47,53^. The biweekly GIMSS-NDVI series were composited monthly, according to the maximum monthly value, to further eliminate disturbance from clouds, atmospheric conditions, and changes in solar altitude angle^26^. In order to match the NDVI data with the climate datasets (0.5° spatial resolution), we averaged the monthly NDVI data that corresponded to each climate dataset pixel.

### Land cover data

We used the 5.1 MODIS land cover type climate modelling grid product (MCD12C1), which had a spatial resolution of 0.05° and provided dominant land cover types from 2001 to 2012^54,55^. We used a land cover classification that was derived from a supervised decision-tree classification method and that was defined by the International Geography Biosphere Programme (IGBP). The classification system included 11 natural vegetation classes, three developed and mosaicked land classes, and three non-vegetated land classes. To match the data with the CRU climate datasets, we used the majority scheme to resample the land cover data to a 0.5° spatial resolution, for the period from 2001 to 2012, as described by Jong *et al*.^47^. Then, to minimize the impacts of land cover changes on vegetation, only pixels that exhibited constant vegetation types from 2001 to 2012 were kept, which yielded an unchanged vegetation type map for 2001 to 2012, at a spatial resolution of 0.5° (Fig. S4). Moreover, due to the apparent differences in climate conditions observed for shrubland at high and low latitudes, shrublands distributed north and south of 45°N were divided into two categories (Table S1).

### Analysis

Because little is known about the general temporal pattern of vegetation water memory length on a monthly scale and global scales^16^, we assumed that the length of water memory would range from 1 to 12 months. All of the time series of the GIMMS NDVI and climate variables were detrended and standardized^10^. We defined the growing season as any month with a mean NDVI larger than 0.1 and a mean monthly temperature of larger than 0 °C. Additionally, pixels with annual mean NDVI values of less than 0.1 during the 31 years were excluded from the analysis. Moreover, to eliminate the negative effects from the missing values, we removed all areas where the percentage of missing values in the CRU precipitation, temperature and cloud cover datasets was greater than 5% based on the station files (Fig. S1). Thus, for each pixel, we obtained N (growing season length) series of NDVI values (one per month for each month of the growing season) from 1982 to 2012, as well as the 1- to 12-month SPI series. Then, to differentiate the responses of vegetation to SPI during different time scales within growing seasons, each of the NDVI time series was correlated with the corresponding 1- to 12-month SPI series for the period from 1982 to 2012. Finally, we obtained N*12 correlation values for each pixel (12 for each month of the growing season), and the time scale at which the maximum correlation coefficient between SPI and NDVI was highest was defined as the length of water memory.

Considering water memory effects, we took past precipitation information into account and constructed a multiple linear regression (MLR) model to quantify the sensitivity of vegetation productivity to climate variability and to identify regions with strong water memory effects, using the following equation:

1 $$NDV{I}_{t}={\rm{\alpha }}SP{I}_{t}+{\rm{\beta }}{T}_{t}+{\rm{\delta }}{R}_{t}+{\rm{\varepsilon }},$$

where *NDVI*_*t*_ is the standardized NDVI anomaly series at moment *t*; *SPI*_*t*_ is the standardized SPI index at moment *t* at the time scale of water memory length; *T*_*t*_ is the standardized temperature anomaly series at moment *t*; *R*_*t*_ is the standardized radiation anomaly series at moment *t*; and *ε* is the residual error. Since the time series of data were detrended and standardized, the model coefficients were comparable. In particular, *SPI* quantifies the variation of within-memory precipitation; α describes the sensitivity of NDVI to changes in *SPI*; and β and δ refer to the sensitivity of NDVI to changes in temperature and shortwave radiation. Positive (negative) values of α, β and δ indicate an increase (decrease) in the NDVI response to the climate under wetter, warmer and higher solar-radiation conditions, respectively. In addition, we also constructed a model without considering antecedent precipitation, in which *SPI* was replaced by the contemporary precipitation, using the following equation:

2 $$NDV{I}_{t}=\alpha 2{P}_{t}+\beta 2{T}_{t}+\delta 2{R}_{t}+\varepsilon 2,$$

where P is the standardized precipitation anomaly series at moment t, and other variables are similar to equation (1).

To investigate the relationship between water memory effects and vegetation memory effects or engineering resilience, we calculated the memory effects or engineering resilience of global vegetation using the following equation:

3 $$NDV{I}_{t}=\gamma NDV{I}_{t-1}+\alpha 3{P}_{t}+\beta 3{T}_{t}+\delta 3{R}_{t}+\varepsilon 3.$$

As described by Keersmaecker *et al*.^4^, *NDVI*_t−1_ is the standardized NDVI anomaly series at moment _t−1_, and γ indicates the magnitude of memory effects and the relative ecosystem recovery rates to an equilibrium state. In general, high absolute values of γ indicated strong memory effects and low recovery rate to equilibrium (i.e., a low engineering resilience). Other variables are similar to equation (1).

### Data availability statement

The datasets generated during and/or analyzed during the current study are available from the corresponding author on reasonable request.

## Electronic supplementary material

Supplementary information
