# Supplementary material for: Water memory effects and their impacts on global vegetation productivity and resilience
Source: Sci Rep. 2018 Feb 13;8:2962. doi: 10.1038/s41598-018-21339-4 (PMC5811601; doi:10.1038/s41598-018-21339-4)
Supplement: Supplementary file 1 — Supplementary information [file 41598_2018_21339_MOESM1_ESM.docx]

**Supplementary information**

**Water memory effects and their impacts on global vegetation productivity and resilience**

Laibao Liu^1,2^, Yatong Zhang^1,2^, Shuyao Wu^1,2^, Shuangcheng Li^1,2*^, Dahe Qin^3^

1 College of Urban and Environmental Sciences, Peking University, Beijing 100871, China.

2 Key Laboratory for Earth Surface Processes of The Ministry of Education, Peking University, Beijing 100871, China.

3 State Key Laboratory of Cryospheric Sciences, Cold and Arid Regions Environmental and Engineering Research Institute, Chinese Academy of Sciences, Lanzhou, China

Corresponding Author**:** scli@urban.pku.edu.cn

**Table S1 | Statistics of the water memory length**

| ID | IGBP Land Cover Type | Mean | SD | N |
| --- | --- | --- | --- | --- |
| 01 | Evergreen needleleaf forest | 4.5 | 3.6 | 1131 |
| 02 | Evergreen broadleaf forest | 5.0 | 3.6 | 1364 |
| 03 | Deciduous needleleaf forest | 5.2 | 4.1 | 728 |
| 04 | Deciduous broadleaf forest | 5.2 | 3.5 | 395 |
| N7 | Open shrubland (North) | 5.9 | 4.2 | 5824 |
| S7 | Open shrubland (South) | 6.9 | 3.1 | 3152 |
| 08 | Woody savanna | 5.1 | 3.5 | 3358 |
| 09 | Savanna | 5.2 | 2.9 | 2699 |
| 10 | Grassland | 6.5 | 3.7 | 6447 |

IGBP, International Geography Biosphere Programme; Mean, the averaged grids values (length of water memory) for each vegetation type; SD, standard deviation; N, number of pixels.

**Table S2 | Statistics of the NDVI_t-1_ coefficients and Mean annual precipitation (MAP)**

| ID | IGBP Land Cover Type | Mean | SD | MAP |  |
| --- | --- | --- | --- | --- | --- |
| 01 | Evergreen needleleaf forest | 0.26 | 0.09 | 840.02 |  |
| 02 | Evergreen broadleaf forest | 0.25 | 0.10 | 2351.89 |  |
| 03 | Deciduous needleleaf forest | 0.28 | 0.10 | 390.17 |  |
| 04 | Deciduous broadleaf forest | 0.32 | 0.12 | 915.22 |  |
| N7 | Open shrubland (North) | 0.35 | 0.13 | 344.94 |  |
| S7 | Open shrubland (South) | 0.59 | 0.11 | 317.54 |  |
| 08 | Woody savanna | 0.33 | 0.13 | 834.91 |  |
| 09 | Savanna | 0.40 | 0.11 | 953.78 |  |
| 10 | Grassland | 0.42 | 0.13 | 448.97 |  |

IGBP, International Geography Biosphere Programme; Mean, the averaged grids values (NDVI_t-1_ coefficient) for each vegetation type; SD, standard deviation; MAP, the averaged grids values (mean annual precipitation) for each vegetation type.


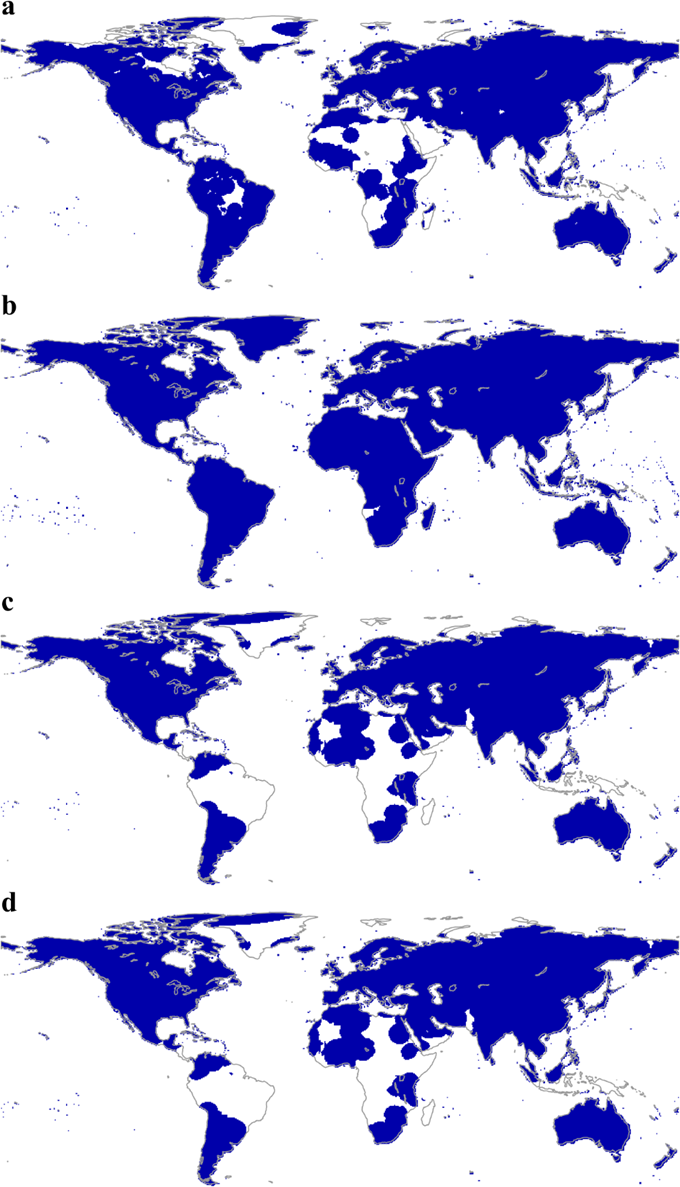


**Figure S1.** **Areas with the percentage of missing values less than 5% in the CRU TS4.01 (a) precipitation dataset during 1960-2013, (b) temperature dataset during 1982-2012, (c) cloud cover dataset during 1982-2012, and (d) precipitation, temperature and cloud cover datasets during the study period (in blue).** The number of missing value in each variable is identified by the CRU TS4.01 station files. Maps were produced using MATLAB R2016b (http://www.mathworks.com/products/matlab/).


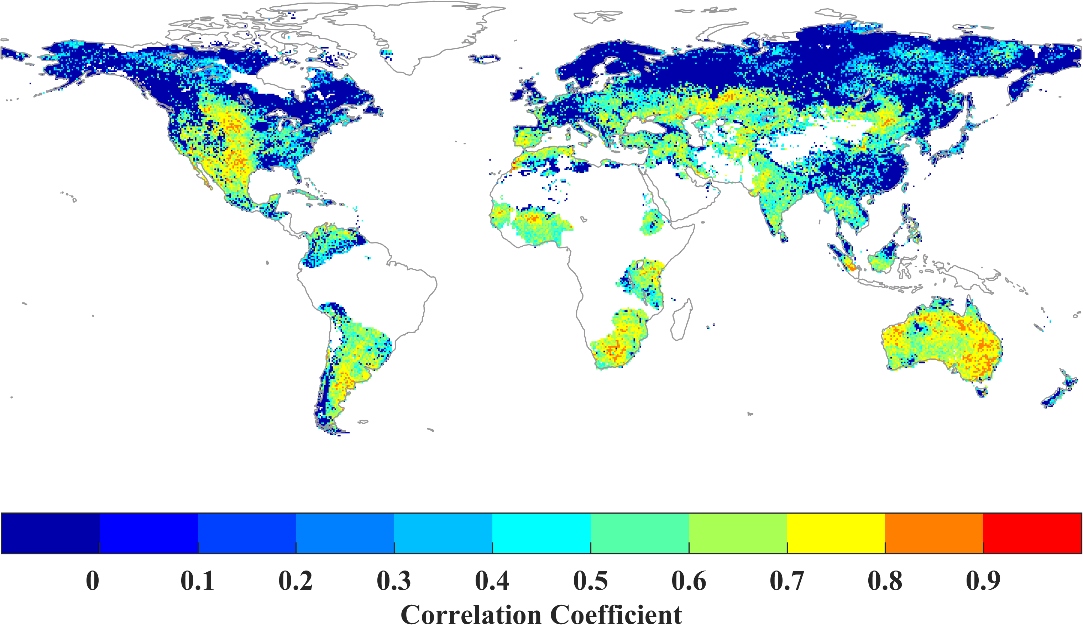


**Figure S2. Spatial distribution of maximum correlation coefficients between Normalized Difference Vegetation Index (NDVI) and Standardized Precipitation Index (SPI) during the growing season from 1982 to 2012**. Areas with barren land (mean NDVI<0.1 for all months), permanent ice, and the percentage of missing values greater than 5% in the CRU TS4.01 climate datasets are not shown. The map was produced using MATLAB R2016b (http://www.mathworks.com/products/matlab/).


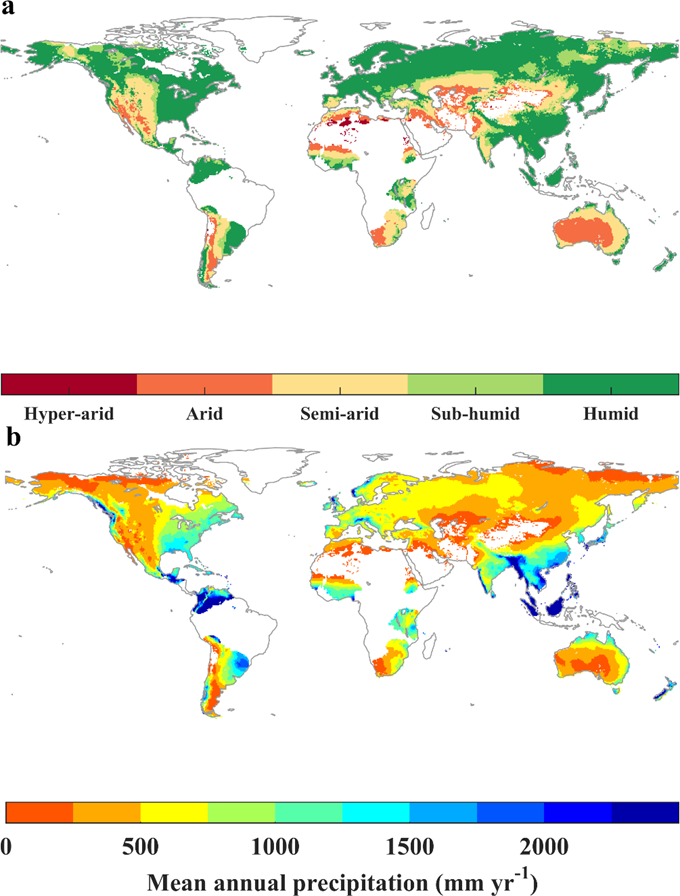


**Figure S3.** **(a) Identification of arid and semi-arid regions based on aridity index**. Aridity index (AI) is the ratio of precipitation to potential evapotranspiration. In general, AI in arid regions range from 0.05 to 0.2, and AI in semi-arid regions range from 0.2 to 0.5. **(b)** **Spatial distribution of mean annual precipitation during 1982-2012 using CRU TS4.01 precipitation data set.** Areas with the percentage of missing values greater than 5% in the CRU TS4.01 climate datasets are not shown. Maps were produced using MATLAB R2016b (http://www.mathworks.com/products/matlab/).


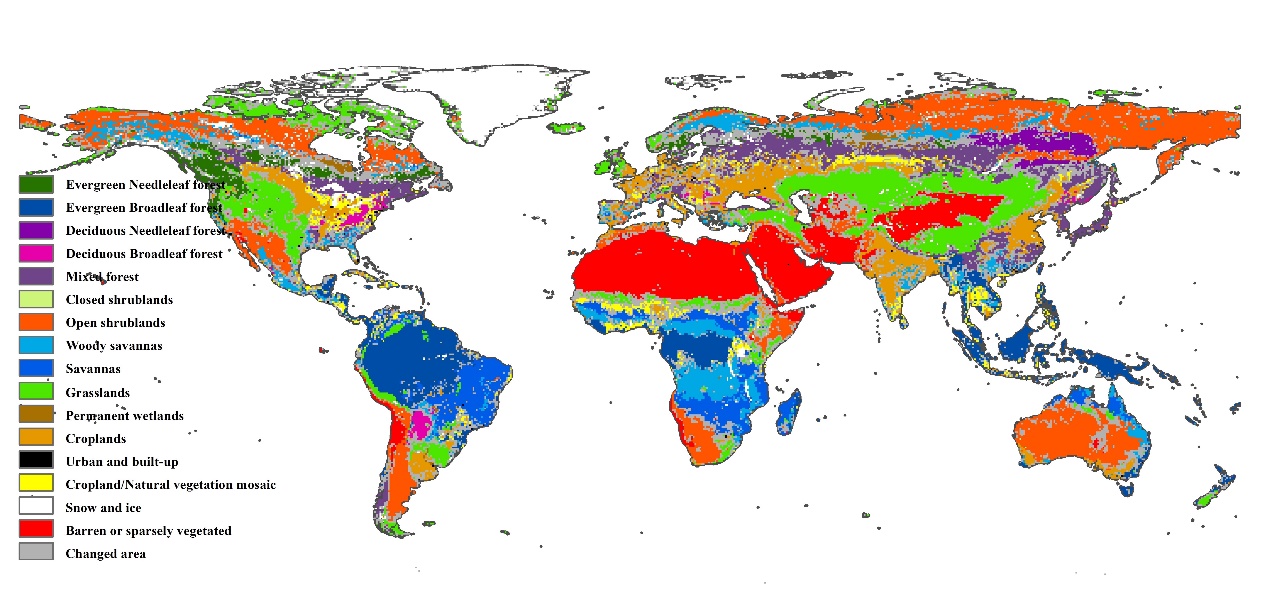


**Figure S4. Map of areas with unchanged land use types from 2001 to 2012, at a spatial resolution of 0.5°.** Data were derived from the 5.1 MODIS land cover type climate modelling grid product (MCD12C1). The map was produced using MATLAB R2016b (http://www.mathworks.com/products/matlab/).


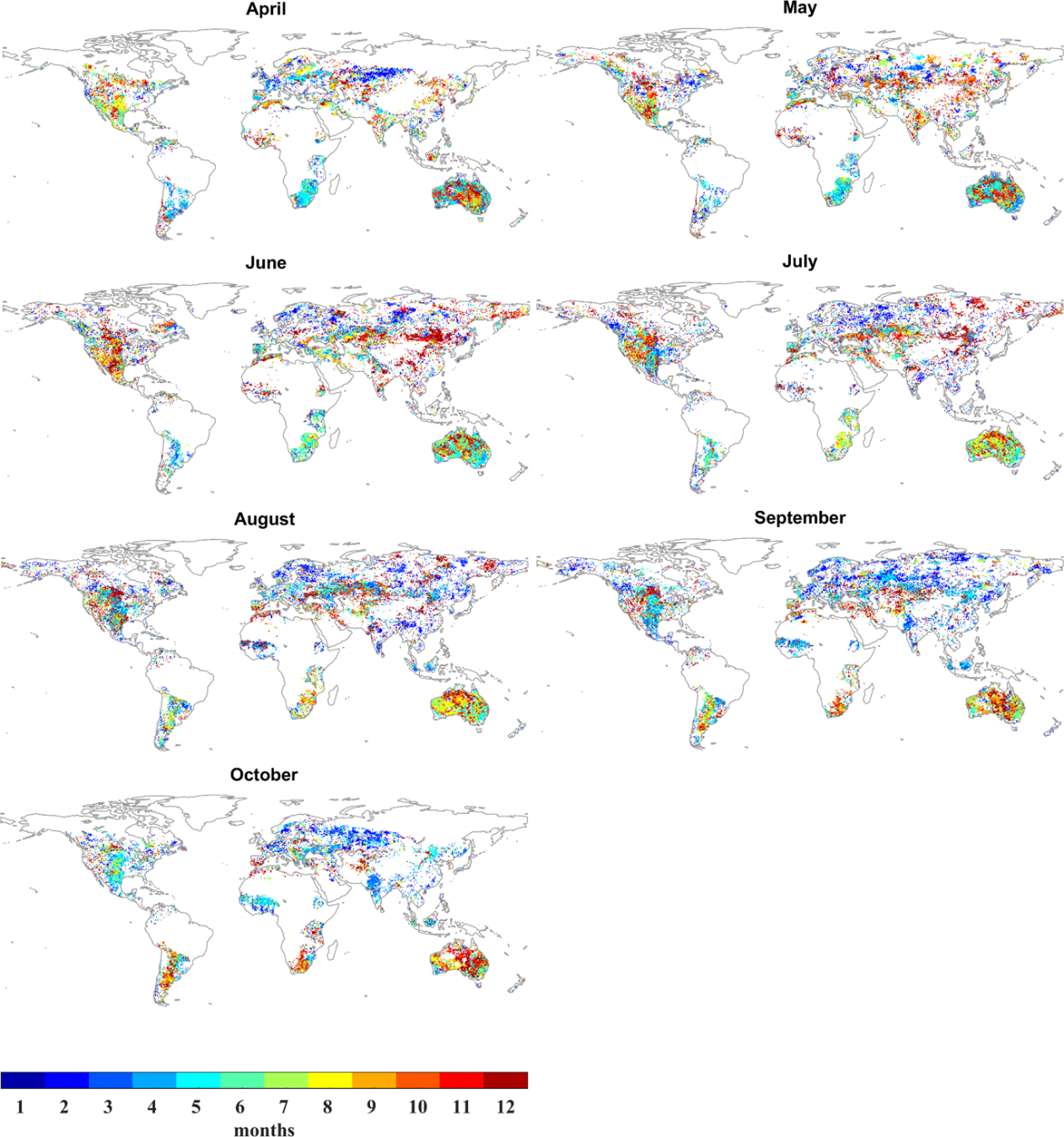


**Figure S5. Spatial distribution of the time scales of at which the correlation coefficients between the Standardized Precipitation Index (SPI) and Normalized Difference Vegetation Index** (**NDVI) was highest in the main growing season (April to October in the NH and October to April in the SH).** Because the growing season of most areas cover April-October, so we just show the results of the April-October. Areas with no significant correlations, barren land (mean NDVI<0.1 for all months), permanent ice, and the percentage of missing values greater than 5% in the CRU TS4.01 climate datasets are not shown. Maps were produced using MATLAB R2016b (http://www.mathworks.com/products/matlab/).


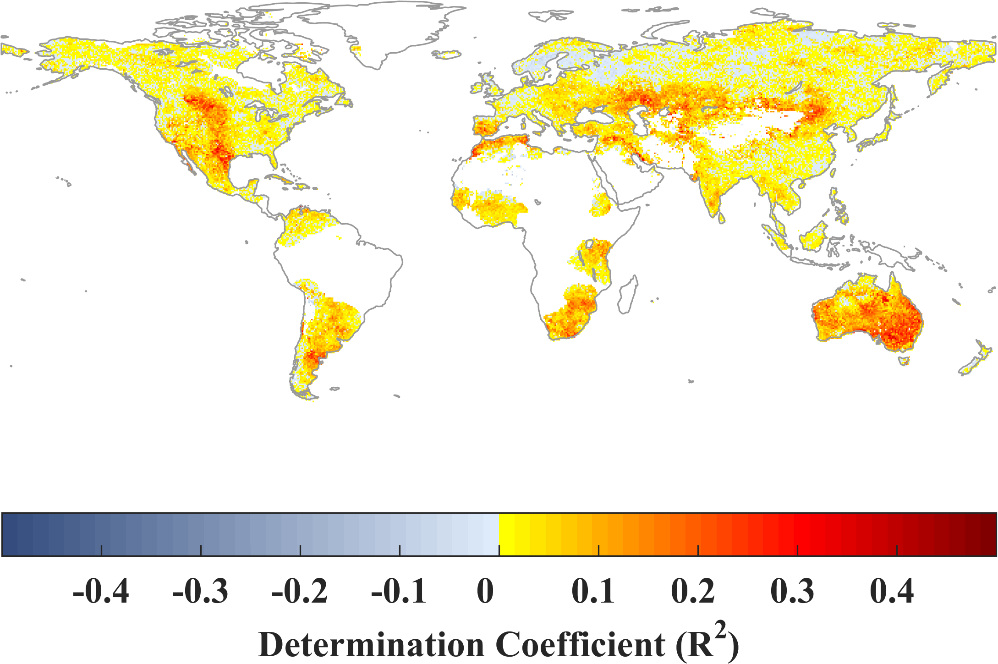


**Figure S6. Spatial distribution of differences in the determination coefficient (R^2^) values of multiple linear regression models with and without considering water memory effect from 1982 to 2012.** Areas with barren land (mean NDVI<0.1 for all months), permanent ice, and the percentage of missing values greater than 5% in the CRU TS4.01 climate datasets are not shown. The map was produced using MATLAB R2016b (http://www.mathworks.com/products/matlab/).


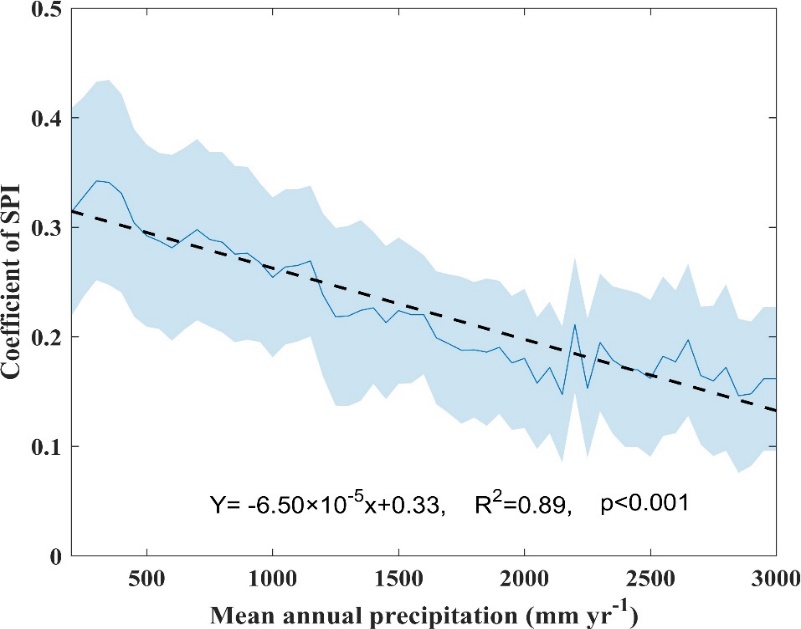


**Figure S7. Relationship between the coefficient of Standardized Precipitation Index (SPI) and mean annual precipitation (MAP) from 1982 to 2012.** Note that the values of the SPI coefficient characterize the sensitivity of the Normalized Difference Vegetation Index (NDVI) to variation in SPI. The solid line and shaded area represent the means ± SD/2. The pixel values of SPI coefficient were averaged by MAP bin (each interval size of 50 mm yr^-1^).The dashed line represents the linear regression of SPI coefficient values from the multiple linear regression model and MAP. The map was produced using MATLAB R2016b (http://www.mathworks.com/products/matlab/).


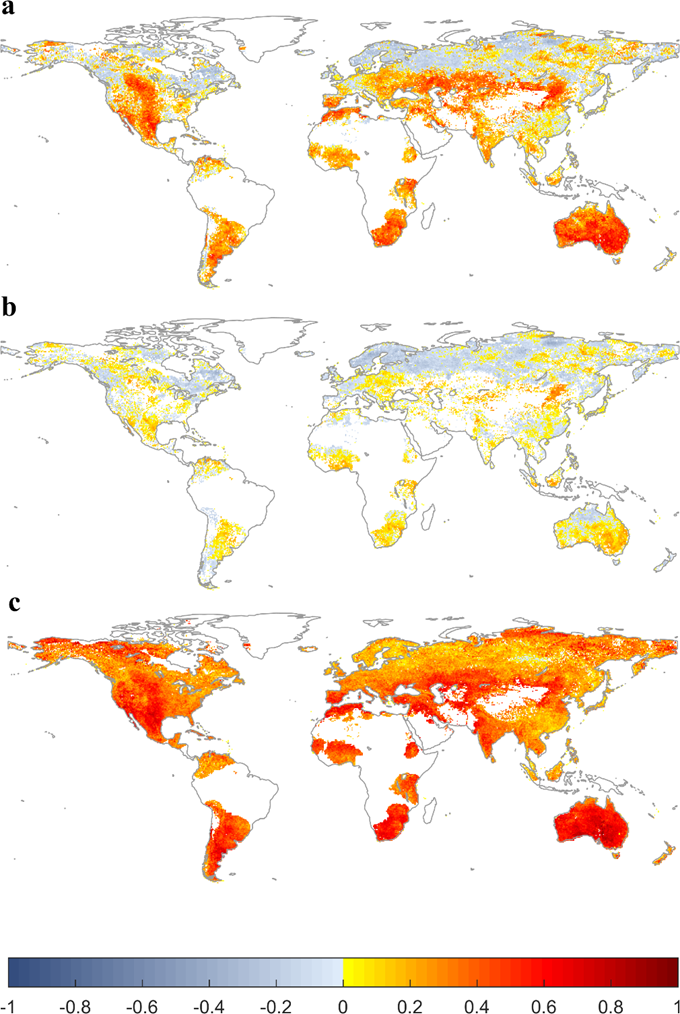


**Figure S8.** **Same as Fig.2, but the SPI and precipitation was substituted by GPCC SPI and precipitation.** The map was produced using MATLAB R2016b (http://www.mathworks.com/products/matlab/).


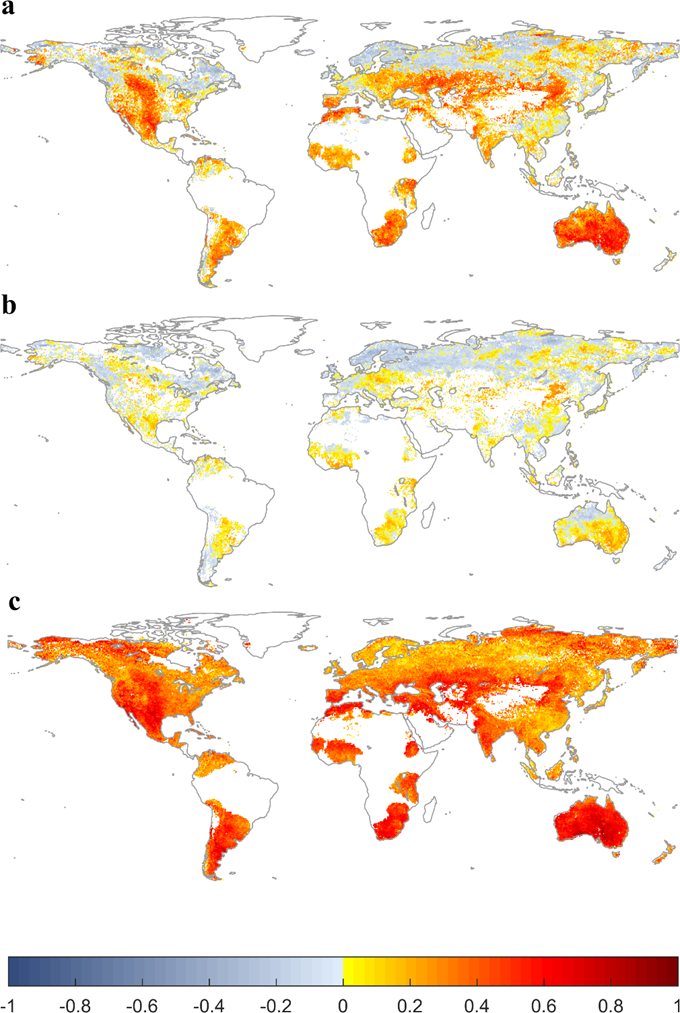


**Figure S9.** **Same as Fig.2, but the SPI and precipitation was substituted by UDel SPI and precipitation.** The map was produced using MATLAB R2016b (http://www.mathworks.com/products/matlab/).


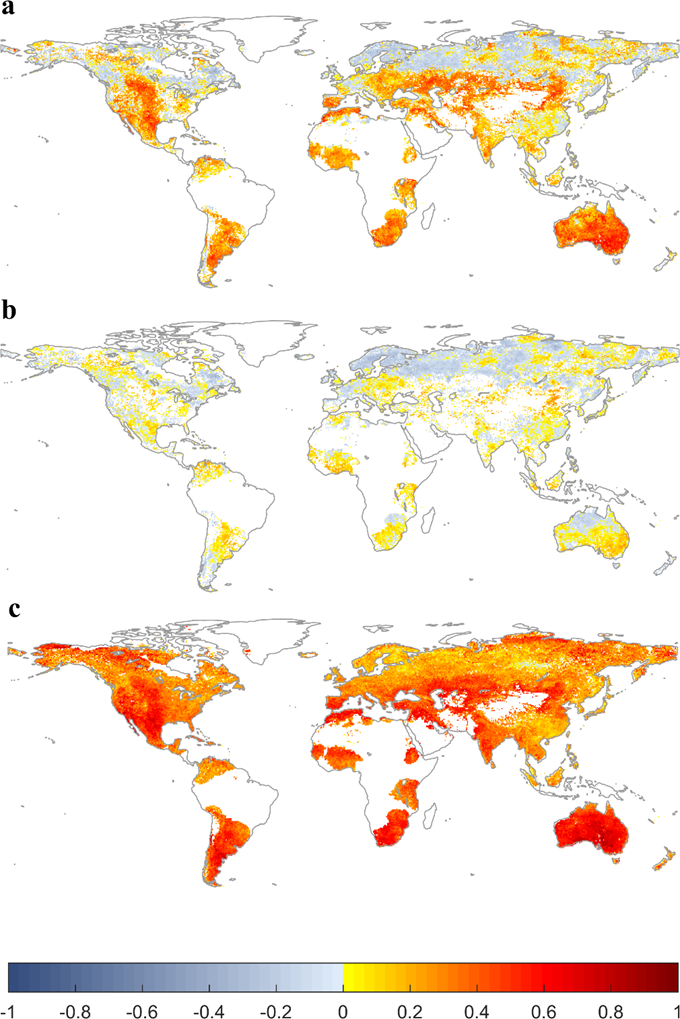


**Figure S10.** **Same as Fig.2, but the** **temperature was substituted by UDel temperature.** The map was produced using MATLAB R2016b (http://www.mathworks.com/products/matlab/).


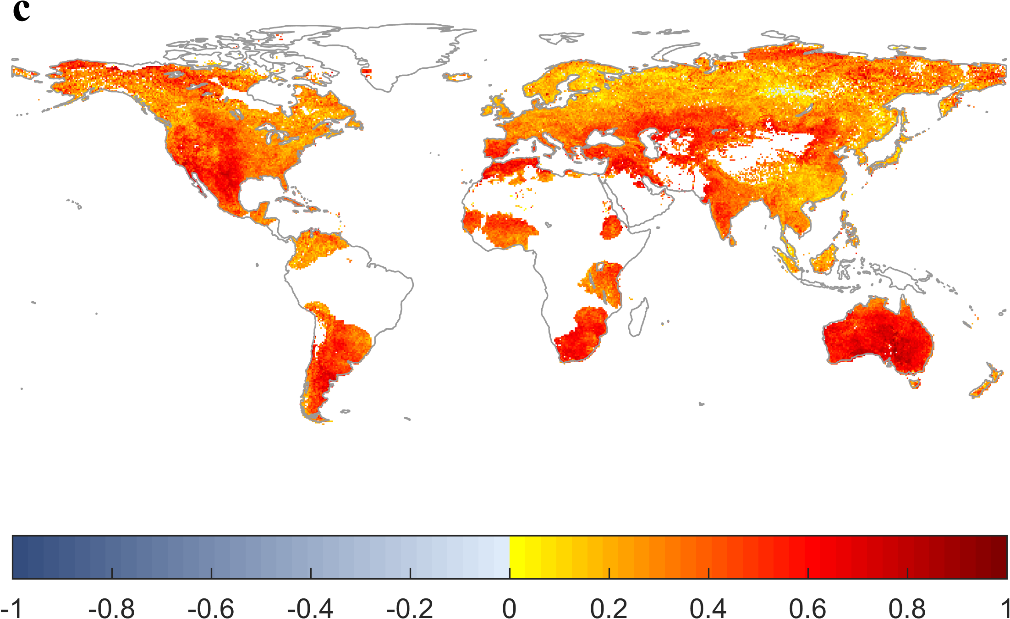

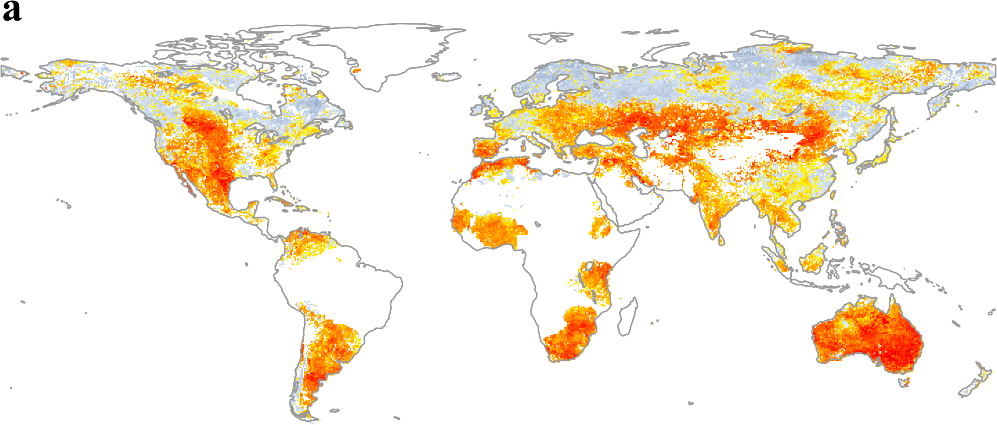

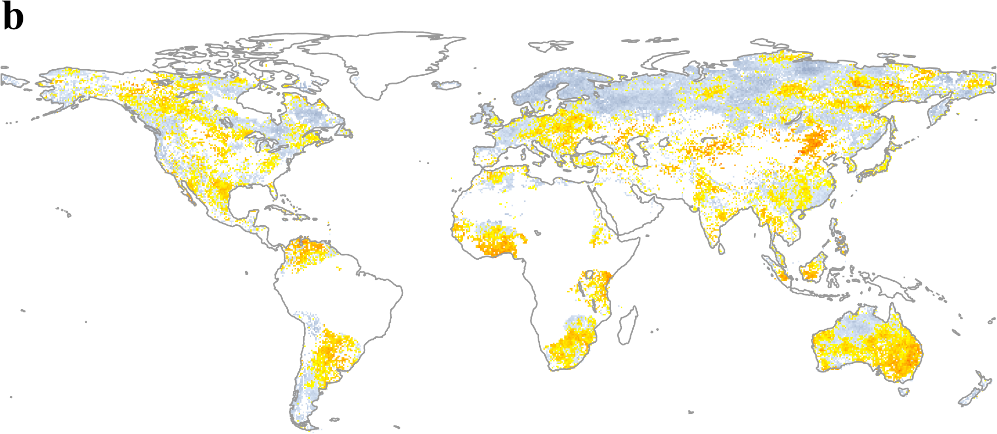


**Figure S11.** **Same as Fig.2, but the cloud cover was substituted by the CRU-NCEP shortwave radiation dataset.** The map was produced using MATLAB R2016b (http://www.mathworks.com/products/matlab/).


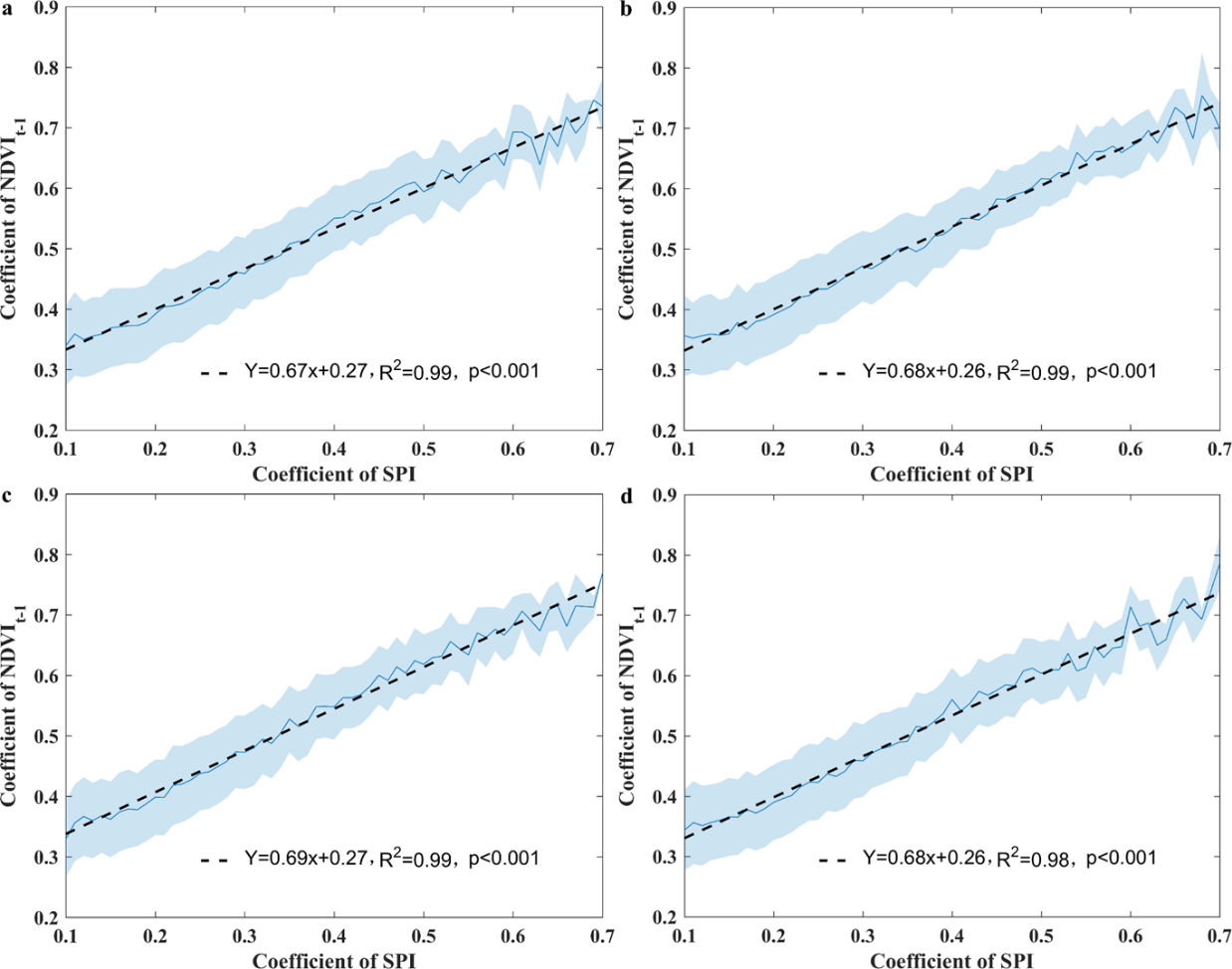


**Figure S12.** **Same as Fig.3, but (a) the SPI and precipitation was substituted by GPCC SPI and precipitation; (b) the SPI and precipitation was substituted by UDel SPI and precipitation; (c) the temperature was substituted by UDel temperature; (d) the** **cloud cover was substituted by the CRU-NCEP shortwave radiation.** The map were produced using MATLAB R2016b (http://www.mathworks.com/products/matlab/).


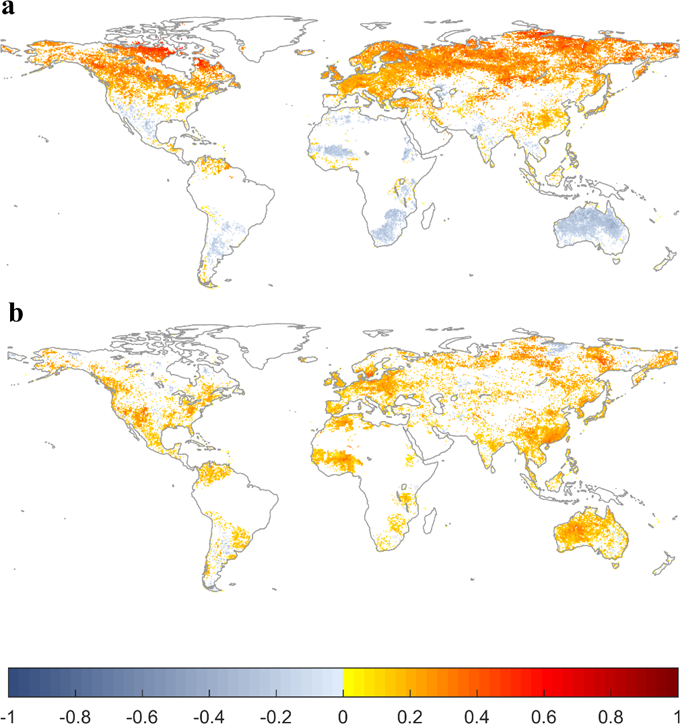


**Figure S13.** **Spatial distribution of (a) temperature coefficients and (b) shortwave radiation coefficients from multiple linear regression model (MLR) considering antecedent precipitation during the growing season from 1982 to 2012.** Areas with no significant relationship (P>0.05), barren land (mean NDVI<0.1 for all months), permanent ice, and the percentage of missing values greater than 5% in the CRU TS4.01 climate datasets are not shown. Maps were produced using MATLAB R2016b (http://www.mathworks.com/products/matlab/).


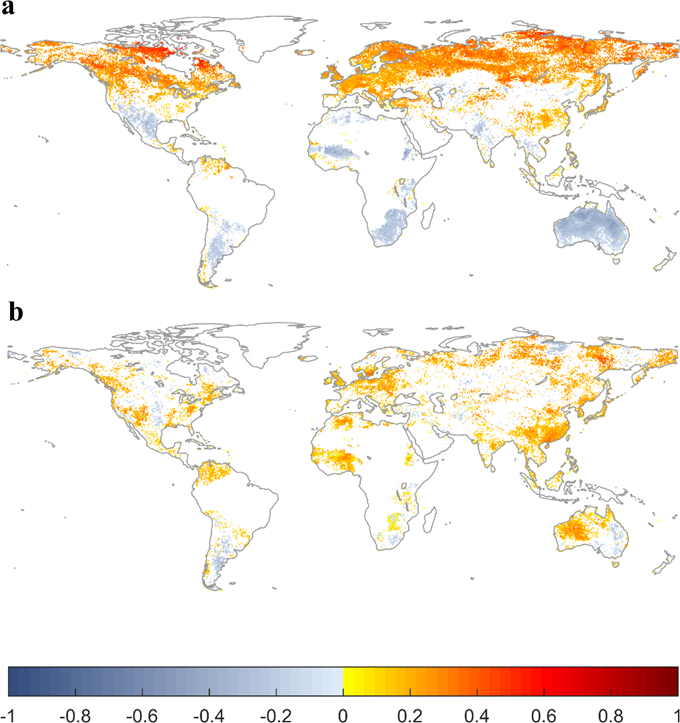


**Figure S14.** **Spatial distribution of (a) temperature coefficients and (b) shortwave radiation coefficients from multiple linear regression (MLR) model without considering antecedent precipitation during the growing season from 1982 to 2012.** Areas with no significant relationship (P>0.05), barren land (mean NDVI<0.1 for all months), permanent ice, and the percentage of missing values greater than 5% in the CRU TS4.01 climate datasets are not shown. Maps were produced using MATLAB R2016b (http://www.mathworks.com/products/matlab/).


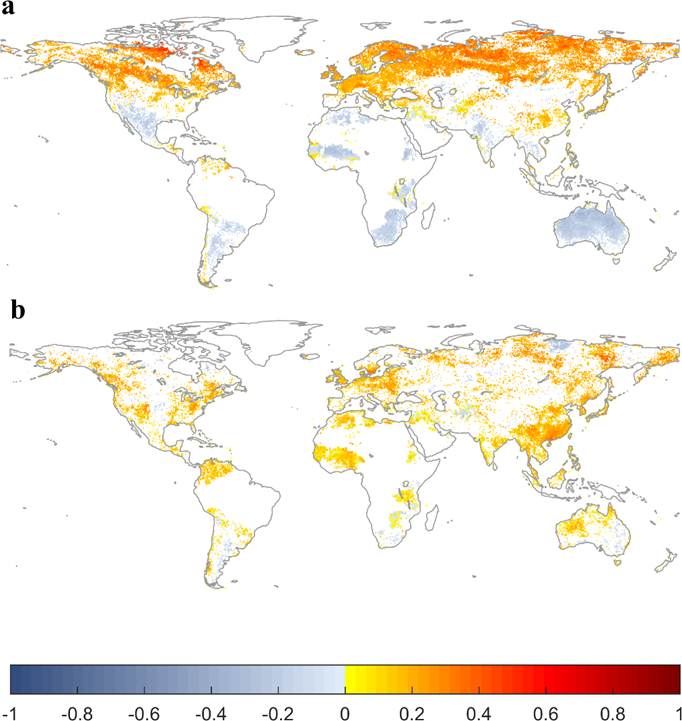


**Figure S15.** **Spatial distribution of (a) temperature coefficients and (b) shortwave radiation coefficients from AR (1) model during the growing season from 1982 to 2012.** Areas with no significant relationship (P>0.05), barren land (mean NDVI<0.1 for all months), permanent ice and the percentage of missing values greater than 5% in the CRU TS4.01 climate datasets are not shown. Maps were produced using MATLAB R2016b (http://www.mathworks.com/products/matlab/).
